# BRD3308 suppresses macrophage oxidative stress and pyroptosis via upregulating acetylation of H3K27 in sepsis-induced acute lung injury

**DOI:** 10.1093/burnst/tkae033

**Published:** 2024-09-02

**Authors:** Bohao Liu, Ning Li, Yi Liu, Yan Zhang, Limei Qu, Hongfei Cai, Yang Li, Xiaojing Wu, Qing Geng

**Affiliations:** Department of Thoracic Surgery, The First Hospital of Jilin University, 71 Xinmin Street, Chaoyang District, Changchun, Jilin, 130021, China; Department of Thoracic Surgery, Renmin Hospital of Wuhan University, 238 Jiefang Road, Wuchang District, Wuhan, Hubei, 430060, China; Department of Thoracic Surgery, Renmin Hospital of Wuhan University, 238 Jiefang Road, Wuchang District, Wuhan, Hubei, 430060, China; Department of Thoracic Surgery, The First Hospital of Jilin University, 71 Xinmin Street, Chaoyang District, Changchun, Jilin, 130021, China; Department of Pathology, The First Hospital of Jilin University, 71 Xinmin Street, Chaoyang District, Changchun, Jilin, 130021, China; Department of Thoracic Surgery, The First Hospital of Jilin University, 71 Xinmin Street, Chaoyang District, Changchun, Jilin, 130021, China; Department of Thoracic Surgery, The First Hospital of Jilin University, 71 Xinmin Street, Chaoyang District, Changchun, Jilin, 130021, China; Organ Transplantation Center, The First Hospital of Jilin University, 71 Xinmin Street, Chaoyang District, Changchun, Jilin, 130021, China; Department of Anesthesiology, Renmin Hospital of Wuhan University, 238 Jiefang Road, Wuchang District, Wuhan, Hubei, 430060, China; Department of Thoracic Surgery, Renmin Hospital of Wuhan University, 238 Jiefang Road, Wuchang District, Wuhan, Hubei, 430060, China

**Keywords:** BRD3308, Acute lung injury, Histone deacetylase 3, Pyroptosis, Reactive oxygen species, Autophagy, Sepsis, Inflammasome, Oxidative stress, Macrophages

## Abstract

**Background:**

Sepsis-induced acute lung injury (ALI) leads to severe hypoxemia and respiratory failure, contributing to poor prognosis in septic patients. Endotoxin dissemination triggers oxidative stress and the release of inflammatory cytokines in macrophages, initiating diffuse alveolar damage. The role of epigenetic histone modifications in organ injury is increasingly recognized. The present study aimed to investigate the use of a histone modification inhibitor to alleviate sepsis-induced ALI, revealing a new strategy for improving sepsis patient survival.

**Methods:**

*In vivo* models of ALI were established through the intraperitoneal injection of lipopolysaccharide and cecal ligation and puncture surgery. Furthermore, the disease process was simulated *in vitro* by stimulating Tamm-Horsfall protein-1 (THP-1) cells with lipopolysaccharide. Hematoxylin and eosin staining, blood gas analysis and pulmonary function tests were utilized to assess the extent of lung tissue damage. Western blot analysis, real-time polymerase chain reaction, enzyme-linked immunosorbent assay and immunofluorescence were used to measure the levels and distribution of the indicated indicators within cells and tissues. Reactive oxygen species and autophagic flux alterations were detected using specific probes.

**Results:**

BRD3308, which is a inhibitor of histone deacetylase 3, improved lung tissue damage, inflammatory infiltration and edema in ALI by inhibiting Nod-like receptor protein3-mediated pyroptosis in macrophages. By upregulating autophagy, BRD3308 improved the disruption of redox balance in macrophages and reduced the accumulation of reactive oxygen species. Mechanistically, BRD3308 inhibited histone deacetylase 3 activity by binding to it and altering its conformation. Following histone deacetylase 3 inhibition, acetylation of H3K27 was significantly increased. Moreover, the increase in H3K27Ac led to the upregulation of autophagy-related gene 5, a key component of autophagosomes, thereby activating autophagy.

**Conclusions:**

BRD3308 inhibits oxidative stress and pyroptosis in macrophages by modulating histone acetylation, thereby preventing sepsis-induced ALI. The present study provides a potential strategy and theoretical basis for the clinical treatment of sepsis-induced ALI.

## Graphical Abstract

**Graphical Abstract:**
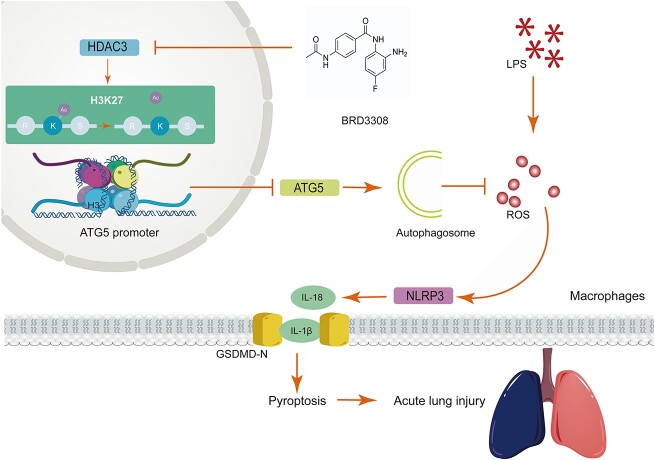

Highlights

- This study reveals for the first time that BRD3308 improves sepsis-induced ALI by promoting H3K27 acetylation through the inhibition of histone deacetylase (HDAC)3 enzymatic activity.
- The study emphasizes the role of HDAC3 in exacerbating inflammatory responses and organ injury by mediating histone deacetylation, which plays a part in regulating oxidative stress in macrophages.
- It elucidates that during ALI, acetylation of H3K27 in macrophages can enhance autophagy to exert protective effects by regulating the expression of the key autophagy protein ATG5.

## Background

Sepsis is one of the most common complications in intensive care unit patients, resulting in a remarkably high mortality rate [1]. The lung, which serves as a crucial site for systemic oxygenation and gas exchange, is highly susceptible to endotoxins that enter the bloodstream. Sepsis-induced acute lung injury (ALI) is one of the most critical factors influencing the prognosis and survival status of septic patients, often leading to acute respiratory distress syndrome and refractory hypoxemia [2]. Despite extensive research efforts in recent years to explore the pathogenesis and preventive strategies of sepsis-induced ALI, mechanical ventilation and symptomatic treatment remain the primary therapeutic modalities in clinical practice.

During sepsis, macrophages, which are crucial regulatory cells of the innate immune system, are recruited in large numbers to lung tissue, where they become activated and release a significant amount of proinflammatory factors and cellular contents [3,4], leading to disruption of the immune microenvironment and diffuse alveolar damage. When lipopolysaccharide (LPS) is recognized by Toll-like receptor 4 on the cell membrane, nuclear factor-κB in macrophages is activated and translocated into the nucleus, leading to the upregulation of Nod-like receptor protein 3 (NLRP3) expression [5]. The NLRP3 inflammasome, which participates in assembly, has dual roles as follows: it promotes the maturation and activation of interleukin (IL)-1β and IL-18; and it cleaves gasdermin-D (GSDMD) and exposes its N-terminus, causing the latter to form pores on the cell membrane [6]. Formation of the pores results in an imbalance of intracellular and extracellular pressure in macrophages, leading to the release of cellular contents and inflammatory cytokines [7]. An increasing body of research indicates that LPS, when used to stimulate macrophages, triggers acute mitochondrial dysfunction and metabolic imbalance, leading to cellular oxidative stress [8,9]. Intracellular aggregation of reactive oxygen species (ROS) both upregulates the transcriptional activity of nuclear factor-κB and facilitates the assembly of the NLRP3 inflammasome, thereby exacerbating pyroptosis [10,11]. In summary, developing drugs that target the inhibition of macrophage pyroptosis by maintaining redox homeostasis and elucidating the related mechanism may offer theoretical support for the prevention and treatment of ALI.

Histone posttranslational modifications epigenetically regulate the expression of the majority of genes in eukaryotic cells by modulating chromatin structure [12]. Histone acetylation, a type of posttranslational modification, leads to the transformation of the originally tightly wound chromatin structure to a more relaxed state, thereby altering the transcription of specific genes at the corresponding DNA loci [13]. Histone deacetylases (HDACs) promote histone deacetylation by removing acetyl groups from lysine residues of histones, which antagonizes the ability of histone acetyltransferases (HATs) to regulate the balance of histone acetylation [13]. In recent years, extensive studies have indicated that HDACs not only promote the onset and progression of tumors but also exacerbate acute and chronic injury to vital organs [14,15]. HDAC3, classified as a class I HDAC, exhibits unrestricted nuclear–cytoplasmic shuttling and regulates both histone and nonhistone deacetylation [16]. Our previous research has demonstrated that in the context of ALI, HDAC3 has a profound impact on mitochondrial quality control in type II alveolar epithelial cells while simultaneously upregulating NLRP3-mediated pyroptosis in macrophages, exacerbating inflammatory responses and alveolar damage [17,18]. HDAC inhibitors (HDACis) are a class of small molecule compounds that target HDACs and inhibit their enzymatic activity by binding to them, thereby either suppressing their catalytic activity or altering their protein stability [19]. Currently, HDACis approved for clinical use have been applied in antitumor therapy, and research has also indicated their protective effects on organ injury [19,20]. Among the known HDACis are RGFP966, RGFP109, entinostat (MS-275) and PD106 [16]. However, there remains a substantial gap in the exploration of the use of HDACis for the prevention and treatment of sepsis-induced ALI. In the present study, we employed BRD3308, a new selective inhibitor of HDAC3, for early intervention in septic mice, observed its potential alleviating effect on ALI, and investigated the associated mechanisms, aiming to provide novel strategies for clinically preventing and treating sepsis-induced ALI.

## Methods

### Animals and treatment

All care and procedures for the experimental animals were conducted in accordance with the guidelines of the National Institutes of Health (USA), and all experimental protocols were approved by the Animal Care and Use Committee of Renmin Hospital, Wuhan University, China. The mice were bred in a specific pathogen-free environment with controlled temperature (24–26°C) and constant humidity (50–60%) under a 12/12-h light/dark cycle with *ad libitum* access to water and food. Male C57BL/6 mice (wild type) were provided by the Hubei Provincial Experimental Animal Center (Wuhan, Hubei Province, China). *Hdac3* floxed mice (*Hdac3*^flox/flox^) were generated on the C57BL/6 background using the CRISPR-Cas9 system by introducing a loxP-inserted fragment between exons 2 and 7, and they were genotyped using specific primers via polymerase chain reaction (PCR) (primer F, GACACCTCTGTGATGAGCCA; and primer R, CCCCTCTTGTCACTTTCCCC). To obtain mice with specific deletion of HDAC3 in macrophages, Lyz2-CreERT2 mice (primer F: GCTGAAGTCCATAGATCGGTAGG; and primer R, TTCTGCGGGAAACCATTTCC) were crossed with *Hdac3*^flox/flox^ mice, and Lyz2-CreERT2^+^-*Hdac3*^flox/flox^ (*Hdac3* cKO) mice were selected as the experimental subjects. Lyz2-CreERT2-*Hdac3*^flox/flox^ (*Hdac3* C) mice from the same litter were used as controls (Figure S1, see online supplementary material). Male mice of the aforementioned genotypes received intraperitoneal injections of tamoxifen (10 mg/kg) for five consecutive days prior to the experiment to induce gene editing.

According to previous reports, an ALI mouse model was generated by intraperitoneal injection of LPS at a dose of 10 mg/kg for 12 h or by performing cecal ligation and puncture (CLP) surgery on the mice for 24 h [21,22]. BRD3308 was administered via intraperitoneal injection at a dose of 5 mg/kg for seven consecutive days prior to modeling [23]. To inhibit autophagy, mice were intraperitoneally injected with 3-MA at a dose of 15 mg/kg for five consecutive days following BRD3308 administration and before modeling. After all interventions were completed, deep anesthesia (0.3% pentobarbital sodium, 0.1 ml/10 g) was administered via intraperitoneal injection, followed by cervical dislocation to euthanize the mice. The left lung tissues of the mice were extracted for subsequent pathological studies, while the right lung tissues were rapidly placed in liquid nitrogen overnight and then transferred to a −80°C ultralow-temperature freezer for molecular biology analysis. Each experimental sample in the group was replicated at least six times.

### Histopathological analysis

The left lung tissues of the mice were fixed in 4% paraformaldehyde solution and subsequently embedded in paraffin. After the paraffin blocks were cut into thin sections of ~3–5 μm, the sections were dewaxed and gradually rehydrated in different concentrations of ethanol. The sections were then subjected to hematoxylin and eosin staining, followed by observation and imaging under an upright microscope. The observer assessed lung injury using a semiquantitative scoring system based on the extent of neutrophil infiltration and pulmonary edema in the lung tissues. The following scoring criteria were used as previously described: 0, minimal damage; 1 to <2, mild damage; 2 to <3, moderate damage; 3 to <4, severe damage; and 4, maximal damage [24].

### Lung wet/dry ratio

After obtaining mouse lung tissue, the surface moisture was gently blotted using absorbent paper. Subsequently, the lung tissue was placed on preweighed clean aluminum foil, and the initial weight was recorded as the wet weight. The sample was then transferred to a 60°C oven until completely dry. The sample was weighed and the weight was recorded as the dry weight. Using the obtained data, the lung wet-to-dry weight ratio was calculated.

### MPO activity assay

Mouse lung tissue was ground into a homogenate at low temperature. The MPO activity of the homogenate was measured using an MPO activity assay kit according to the manufacturer’s instructions.

### Airway resistance and pulmonary ventilation measurement

After acclimating the treated mice for 20 min, mouse airway resistance and pulmonary ventilation postintervention were measured and calculated using a noninvasive small animal respiratory function testing system to evaluate changes in mouse pulmonary function.

### Mouse blood gas analysis

Arterial blood samples were collected from the treated mice and promptly analyzed using an automated blood gas analyzer to measure arterial blood oxygen partial pressure and carbon dioxide partial pressure.

### Assessment of oxidative stress in lung tissue

The protein concentration of the lung tissue homogenate was standardized. The activities of SOD, NADPH oxidase and CAT in the standardized tissue homogenate were measured using the respective assay kits following the manufacturer's instructions. According to the manufacturer’s instructions, MDA levels were determined using an assay kit to analyze the supernatant obtained from preweighed lung tissue.

### Immunofluorescence staining

Blank lung tissue sections were deparaffinized and rehydrated after baking. Subsequently, antigen retrieval was performed, followed by incubation with primary antibodies against NLRP3 and CD68. This step was followed by coincubation with FITC and Cy3-conjugated secondary antibodies. Next, the sections were counterstained with DAPI to label the nuclei, and they were then observed and imaged under a fluorescence inverted microscope.

### Rapid isolation of alveolar macrophages

After undergoing different treatments, the anesthetized mice were immediately subjected to tracheostomy. In brief, 1 ml of cold PBS was slowly injected into the lungs and aspirated, and this process was repeated three times. The collected bronchoalveolar lavage fluid was passed through a 100 μm cell strainer to remove larger debris. The filtered cell suspension was then centrifuged at a low speed to obtain alveolar macrophages. The collected cells were resuspended in DMEM supplemented with 10% heat-inactivated FBS. After allowing the cells to adhere for 30 min, they were washed with PBS for subsequent analysis.

### Cell culture

THP-1 cells were obtained from Procell Life Science & Technology Co., Ltd (Wuhan, China) and were cultured in RPMI-1640 medium supplemented with 10% FBS. Prior to intervention, THP-1 cells were induced to differentiate into macrophages by treatment with 100 ng/ml PMA for 12 h. To mimic the stimulation of macrophages during ALI in the *in vitro* experiments, differentiated THP-1 cells were stimulated with 1 μg/ml LPS for 6 h, treated with 30 nM BRD3308 for 12 h and then treated with 300 μM H_2_O_2_ for 6 h to induce oxidative stress. The autophagy inhibitor 3-MA (5 mM) was added 6 h prior to LPS intervention. si-Autophagy-related gene 5 (ATG5) (50 nmol/l) was transfected into induced THP-1 cells using Lipo2000 for 6 h to silence *Atg5*, followed by a 12-h incubation. Relevant *in vitro* experiments were conducted when the cells reached ~70–90% confluence.

The A549 human alveolar epithelial cell line was obtained from Procell Life Science & Technology Co., Ltd (Wuhan, China). After starvation in serum-free 1640 medium for 6 h, A549 cells were further cultured in conditioned medium from THP-1 cells stimulated with BRD3308 and LPS for 24 h for subsequent analysis.

### Cell viability assay

THP-1 or A549 cells were resuspended and evenly dispensed, with an equal amount added to each well of a 96-well plate. After grouping and treatment, cell viability was assessed using a CCK-8 assay kit according to the manufacturer’s instructions and relevant data were recorded using a microplate reader.

### Apoptosis detection

A549 cells were harvested using trypsin lacking EDTA and washed with prechilled PBS. Under subdued light conditions, annexin V-FITC and 7-AAD PC5.5-A dyes were added to the cells. After a 15-min incubation at room temperature, the fluorescence intensity in each channel was detected using a flow cytometer. The cell concentration for each group was maintained at 1–5 × 10^6^ cells/ml.

### Western blot analysis

The tissue homogenates or cell suspensions containing RIPA lysis buffer were thoroughly lysed at low temperature, and the lysates were quantified and standardized for protein content using the BCA method. Subsequently, the protein suspensions were denatured and subjected to protein electrophoresis on SDS-PAGE gels, followed by transfer to PVDF membranes. After 3 h of blocking, the membranes were incubated with primary antibodies overnight at 4°C, followed by incubation with secondary antibodies at room temperature. The protein bands were visualized and imaged, and the results were analyzed using ImageJ software.

### Real-time PCR

The ground lung tissue or cell pellet was resuspended in TRIzol reagent, followed by stepwise extraction and purification to obtain total RNA. The RNA samples were reverse transcribed into cDNA using a SweScript RT I First Strand cDNA Synthesis Kit (G3331–100, Servicebio, Wuhan, China). The resulting cDNA was amplified using SYBR® Green Real-time PCR Master Mix (QPK-201, TOYOBO, Japan) and the corresponding mRNA expression levels were detected. The primers used in the present study are listed in Table 1.

**Table 1:** Primers used in real-time polymerase chain reaction

**Gene**	**Species**	**Forward primer**	**Reverse primer**
GAPDH	Mouse	ACTCCACTCACGGCAAATTC	TCTCCTATGGTGGTGACGACA
GAPDH	Human	GGGCTGCTTTTAACTCTGGT	TGGCAGGTTTTTCTAGCGG
IL-6	Mouse	CCCCAATTTCCAATGCTCTCC	CCCCAATTTCCAATGCTCTCC
TNF-α	Mouse	ACTGAACTTCGGGGTGATCGGT	TGGTTTGCTACGACGTGGGCTA
IL-1β	Mouse	AATGAAGGAACGGAGGAGCC	CTCCAGCCAAGCTTCCTTGT
MCP1	Mouse	GGATCGGAACCAAATGAGAT	ATTTACGGGTCAACTTCACA
iNOS	Human	TTCAGTATCACAACCTCAGCAAG	TGGACCTGCAAGTTAAAATCCC
COX2	Human	GGCAAATGGGTTTTCAAGATCC	CCATGATTAATACCACAAATTTCACTAC
NLRP3	Human	ATGTGGGGGAGAATGCCTTG	TTGTCTCCGAGAGTGTTGCC

*GAPDH* glyceraldehyde-3-phosphate dehydrogenase, *IL* interleukin, *TNF-α* tumour necrosis factor-α, *MCP-1* monocyte chemoattractant protein-1, *iNOS* inducible nitric oxide synthase, *COX-2* cyclooxygenase-2, *NLRP3* Nod-like receptor protein 3

### Detection of IL-18 and IL-1β levels

The lung tissue was homogenized in prechilled PBS and the supernatant was obtained after centrifugation. For processed cell samples, the culture medium supernatant was collected immediately. The supernatant was then assayed using ELISA kits according to the manufacturer's instructions to measure the levels of IL-18 and IL-1β.

### PI and Hoechst staining

Treated cells were fixed with 4% paraformaldehyde for 20 min. Subsequently, the cells were stained separately with PI (G1021, Servicebio, Wuhan, China) and Hoechst 33258 (G1011, Servicebio, Wuhan, China) for 10 min. After thorough washing under subdued light conditions, the cells were photographed using a fluorescence microscope and the data were recorded.

### Measurement of ROS levels

DCFH-DA probe was added to the culture medium of THP-1 cells at various ratios, followed by incubation for 20 min. Excess probe was removed by washing with serum-free culture medium, and the cells were observed and photographed within 30 min using an inverted fluorescence microscope.

### Transduction of mRFP-GFP-LC3 dual-tagged lentivirus

THP-1 cells, not induced by PMA, were seeded in 6-well plates. A lentivirus carrying mRFP-GFP-LC3 (HB-LP210, Hanbio, Wuhan, China) was added at an MOI of 80. The plate was then centrifuged slowly for 50 min. The next day, the virus-containing medium was discarded and purified concentration-gradient puromycin was added to select transduced THP-1 cells. After the selection was completed, the THP-1 cells were subjected to treatments, stained with DAPI for nuclear labeling and observed under a confocal microscope. Data analysis was performed using ImageJ.

### Molecular docking

Molecular docking was employed to assess the binding affinity of BRD3308 with HDAC3 protein and to predict binding sites. The structure of BRD3308 was retrieved from the PubChem database, converted into a 3D structure and subjected to energy minimization using AutoDock Vina software. The structure of the HDAC3 protein was obtained from previous studies [25]. After preprocessing with PyMOL software, BRD3308 was selected as the ligand, and the HDAC3 protein was selected as the receptor for molecular docking using AutoDock Vina software. The displayed result represents the optimal binding conformation with the lowest binding energy.

### Chromatin immunoprecipitation assay

Following stimulation with BRD3308 or DMSO, THP-1 cells were cross-linked with formaldehyde to facilitate protein–chromatin interactions. Flexible lysis buffer was used to release chromatin and proteins from the THP-1 cells. Specific primary antibodies against HDAC3 or H3K27Ac, along with a negative control IgG antibody, were added to the lysis buffer to promote the formation of antibody–protein–chromatin complexes. The washed antibody–protein–chromatin complexes were then subjected to heat treatment, and the DNA was dissociated using proteinase and DNA extraction reagents. Subsequently, PCR was employed to analyze the degree of binding between HDAC3 or H3K27Ac and the *Atg5* promoter (primer F, CACTTCCGCCCTCTGGTATC; and primer R, GAGGGTGACTGGACTTGTGG).

### Statistical analysis

All data are presented as the mean ± standard deviation. Statistical analyses were performed using SPSS 23.0 and graphs were generated using GraphPad Prism 8. For comparisons involving two groups influenced by a single factor, Student’s t test was utilized for the analysis of normally distributed data with homogeneous variances, whereas the Mann–Whitney U test was used otherwise. For comparisons involving more than two groups influenced by a single factor, one-way analysis of variance was employed, followed by Tamhane's T2 *post hoc* analysis for multiple comparisons. Data involving two-factor interventions were analyzed using two-way analysis of variance, with Sidak’s test for within-group differences and Tukey’s test for between-group differences. Statistical significance was set at *p* < 0.05.

## Results

### BRD3308 significantly alleviates lung injury and respiratory dysfunction in septic mice

**Figure 1:**
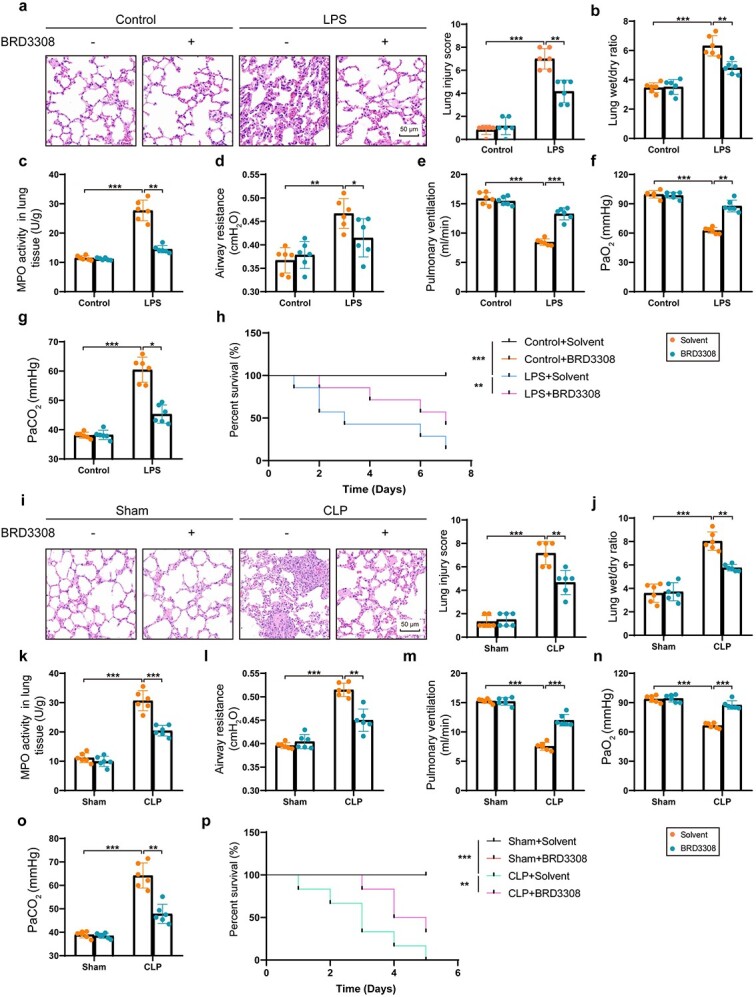
BRD3308 significantly alleviated lung injury and respiratory dysfunction in septic mice. (**a**) Hematoxylin and eosin (H&E) staining images and injury scores of lung tissue sections corresponding to mice in the intraperitoneally injected lipopolysaccharide (LPS) modeling groups with or without BRD3308 treatment (n = 6, Scale bar: 50 μm). (**b**) Lung wet-to-dry ratio was obtained to assess the degree of pulmonary edema after weighing the wet and dry weights of the lung tissues from corresponding groups of mice (n = 6). (**c**) MPO activity in lung tissue was derived from mice in the intraperitoneally injected LPS modeling groups with or without BRD3308 treatment (n = 6). (**d**,**e**) Pulmonary function was assessed based on airway resistance and pulmonary ventilation volume in the indicated groups of mice (n = 6). (**f**,**g**) Blood gas analysis results for the indicated groups (n = 6). (**h**) Kaplan–Meier survival analysis was conducted for mice in respective groups post-injection with PBS or LPS (n = 6, Scale bar: 50 μm). (**i**) H&E staining images and injury scores of lung tissue sections corresponding to mice modelled by cecal ligation and puncture (CLP) surgery with or without BRD3308 treatment (n = 6). (**j**) Lung wet-to-dry ratio was obtained to assess the degree of pulmonary edema after weighing the wet and dry weights of the lung tissues from corresponding groups of mice (n = 6). (**k**) MPO activity in lung tissue was derived from mice in the intraperitoneally injected LPS modeling groups with or without BRD3308 treatment (n = 6). (**l**,**m**) Pulmonary function was assessed based on airway resistance and pulmonary ventilation volume in the indicated groups of mice (n = 6). (**n**,**o**) Blood gas analysis results for the indicated groups (n = 6). (**p**) Kaplan–Meier survival analysis was conducted for mice in respective groups modelled by CLP surgery (n = 6). The data was presented as mean ± standard deviation (^*^p < 0.05, ^*^^*^p < 0.01 or ^*^^*^^*^p < 0.001 compared with indicated group). *MPO *myeloperoxidase, *PBS* Phosphate-Buffered Saline

BRD3308 (5 mg/kg) was administered to wild-type mice via intraperitoneal injection for seven consecutive days. On day 7, sepsis was induced by intraperitoneal injection of LPS or through CLP surgery. As shown in Figure 1a, sepsis led to severe damage and the infiltration of proinflammatory cells into the lung tissue of mice, whereas the administration of BRD3308 markedly reversed this phenomenon. Concurrently, the pulmonary edema induced by sepsis was also ameliorated (Figure 1b). MPO activity in pulmonary tissue contributes to the degree of neutrophil infiltration during the inflammatory response. Early intervention with BRD3308 effectively suppressed sepsis-induced neutrophil infiltration (Figure 1c). As shown in Figure 1d and e, pulmonary function, as assessed through airway resistance and pulmonary ventilation, was significantly compromised by LPS but restored by BRD3308. Blood gas analysis revealed that mice injected with BRD3308 exhibited increased oxygen partial pressure and decreased CO_2_ retention following LPS stimulation (Figure 1f and g). In addition to the above effects, pretreatment with BRD3308 significantly improved survival in mice following the onset of ALI (Figure 1h). In septic mice subjected to CLP, BRD3308 had a similar protective effect on lung tissue (Figure 1i–p).

### BRD3308 reduces the level of inflammation and pyroptosis in alveolar macrophages in sepsis-induced ALI

**Figure 2:**
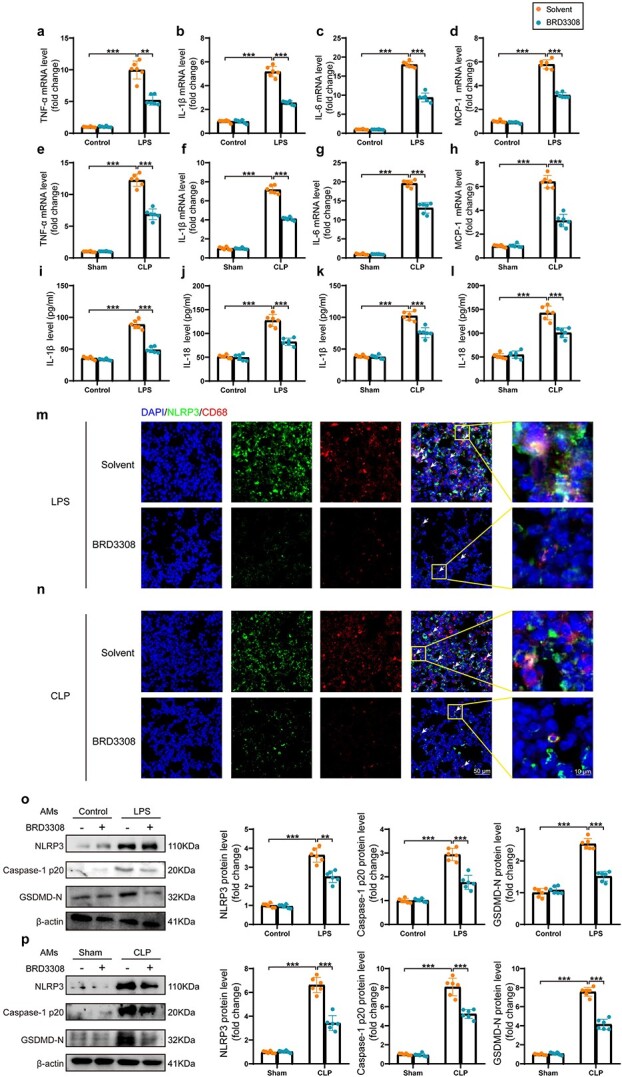
BRD3308 reduced the level of inflammation and pyroptosis of alveolar macrophages in sepsis-induced acute lung injury. mRNA levels of TNF-α, IL-1β, IL-6 and MCP-1 in mouse lung tissues, which were modeled by LPS injection (**a**–**d**) and cecal ligation and puncture (CLP) surgery (**e**–**h**) and subjected to different treatments, were obtained through real-time polymerase chain reaction detection (n = 6). (**i–l**) Activity levels of IL-1β and IL-18 in lung tissue were determined by ELISA (n = 6). (**m**,**n**) Expression and distribution of NLRP3 and CD68 in lung tissue, as revealed by immunofluorescence staining images (n = 6, Scale bar: 50 μm & 20 μm). (**o**,**p**) Western blots images and data analysis to demonstrate the expression levels of NLRP3, caspase-1 p20 and GSDMD-N in treated mouse alveolar macrophages (n = 6). The data are presented as mean ± standard deviation (^*^^*^p < 0.01 or ^*^^*^^*^p < 0.001 compared with indicated group).* TNF-α* tumor necrosis factor α, *ELISA *enzyme-linked immunosorbent assay, *IL* interleukin, *NLRP3* Nod-like receptor protein 3, *GSDMD* gasdermin-D, *LPS* lipopolysaccharide, *MCP-1* monocyte chemoattractant protein-1

Detecting the mRNA levels of inflammatory cytokines and proinflammatory chemokines can sensitively and accurately reflect the level of inflammation in mouse lung tissue in response to stimuli. As shown in Figure 2a–h, the transcription levels of TNF-α, IL-1β, IL-6 and MCP-1 were assessed in lung tissue using real-time PCR. The measurement of TNF-α and IL-6 levels in lung tissue by ELISA revealed that BRD3308 mitigated the release of inflammatory cytokines (Figure S2, see online supplementary material), which indicated that BRD3308 had a significant anti-inflammatory effect on sepsis-induced ALI. LPS significantly upregulated the secretion of IL-18 and IL-1β in mouse lung tissue, indicating pyroptosis. Moreover, BRD3308 reversed the activation and secretion of both genes (Figure 2i–l). An increasing number of studies have suggested that pyroptosis plays an indispensable role in sepsis-induced organ injury. Regardless of whether the mice were stimulated with LPS or subjected to CLP, immunofluorescence analysis indicated that septic mice exhibited a significant increase in macrophage infiltration in lung tissues, which was accompanied by elevated expression of NLRP3 within macrophages (Figure 2m and n). However, in mice that were administered BRD3308 and subsequently challenged with sepsis, both macrophage infiltration and NLRP3 expression were markedly suppressed (Figure 2m and n). To validate the fundamental function of BRD3308 in improving ALI *in vivo*, alveolar macrophages were isolated from mice. As shown in Figures 2o and p, the protein levels of pyroptosis markers, including NLRP3, caspase-1 p20 and GSDMD-N, were suppressed by BRD3308, which indicated that BRD3308 may alleviate sepsis-induced ALI by inhibiting pyroptosis in macrophages.

### BRD3308 inhibits NLRP3-mediated pyroptosis in THP-1 cells and its pro-apoptotic effect on epithelial cells upon LPS stimulation

**Figure 3:**
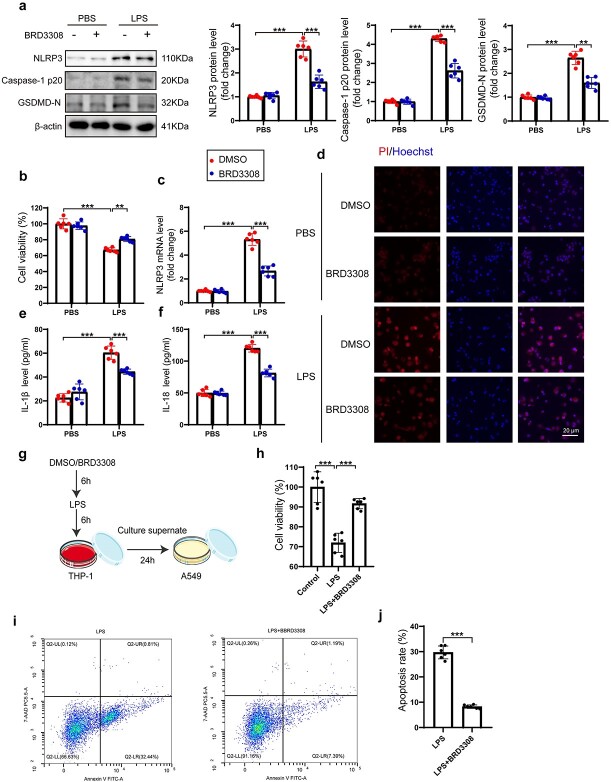
BRD3308 inhibited NLRP3-mediated pyroptosis of THP-1 cells and its pro-apoptotic effect on epithelial cells upon LPS stimulation. (**a**) Western blots images and data analysis to demonstrate the expression levels of NLRP3, caspase-1 p20 and GSDMD-N in THP-1 cells (n = 6). (**b**) Cell viability of THP-1 cells assessed by CCK-8 assay (n = 6). (**c**) mRNA level of NLRP3 in THP-1 cells was obtained through real-time PCR detection (n = 6). (**d**) Representative images of PI & Hoechst staining in THP-1 cells from the indicated groups (n = 3, Scale bar: 20 μm). (**e**,**f**) Activity levels of IL-1β and IL-18 in cell supernatant were determined by ELISA (n = 6). (**g**) THP-1 cell culture medium collected after LPS stimulation with or without BRD3308 treatment was used to intervene with A549 cells, and the extent of damage in the latter after 24 h was assessed to evaluate the protective effect of BRD3308 on epithelial cells. (**h**) Cell viability of A549 cells assessed by CCK-8 assay (n = 6). (**i**,**j**) The degree of apoptosis in A549 cells was assessed using flow cytometry (n = 6). The data are presented as mean ± standard deviation (^*^^*^p < 0.01, ^*^^*^^*^p < 0.001 compared with indicated group). *THP-1* Tamm-Horsfall protein-1, *LPS* lipopolysaccharide, *ELISA* enzyme-linked immunosorbent assay, *IL* interleukin, *NLRP3* Nod-like receptor protein 3, *GSDMD* gasdermin-D

To further explore the related function and specific mechanism of BRD3308 in macrophage pyroptosis, an *in vitro* model was established using LPS-stimulated THP-1 cells. The protein levels of pyroptosis markers in THP-1 cells pretreated with BRD3308 for 0.5 h and stimulated with LPS were assessed by western blot analysis. A significant increase in the levels of NLRP3, caspase-1 p20 and GSDMD-N was detected after LPS stimulation. However, in THP-1 cells pretreated with BRD3308, the expression levels of these three markers were markedly reduced (Figure 3a). Moreover, BRD3308 effectively increased the viability of THP-1 cells upon LPS stimulation (Figure 3b). Consistent with the above results, the transcriptional level of NLRP3 was significantly reduced under the influence of BRD3308, providing a potential direction for further exploration of its mechanism of action (Figure 3c). PI and Hoechst staining, which provide a visual assessment of the extent of pyroptosis, reflect both cell membrane damage and DNA injury. Figure 3d shows that the membrane damage in LPS-induced THP-1 cells was significantly reversed by BRD3308. The activation and secretion of IL-1β and IL-18, induced by pyroptosis, could also be suppressed by BRD3308 (Figure 3e–f). Conditioned medium from processed THP-1 cells was used to culture starved A549 epithelial cells (Figure 3g). Compared to the LPS group, the LPS + BRD3308 group showed enhanced cell viability and reduced levels of apoptosis (Figure 3h–j). These findings further confirmed that BRD3308 inhibits NLRP3-mediated macrophage pyroptosis during sepsis to preserve the integrity of the alveolar epithelium.

### BRD3308 downregulates macrophage pyroptosis by inhibiting oxidative stress

**Figure 4:**
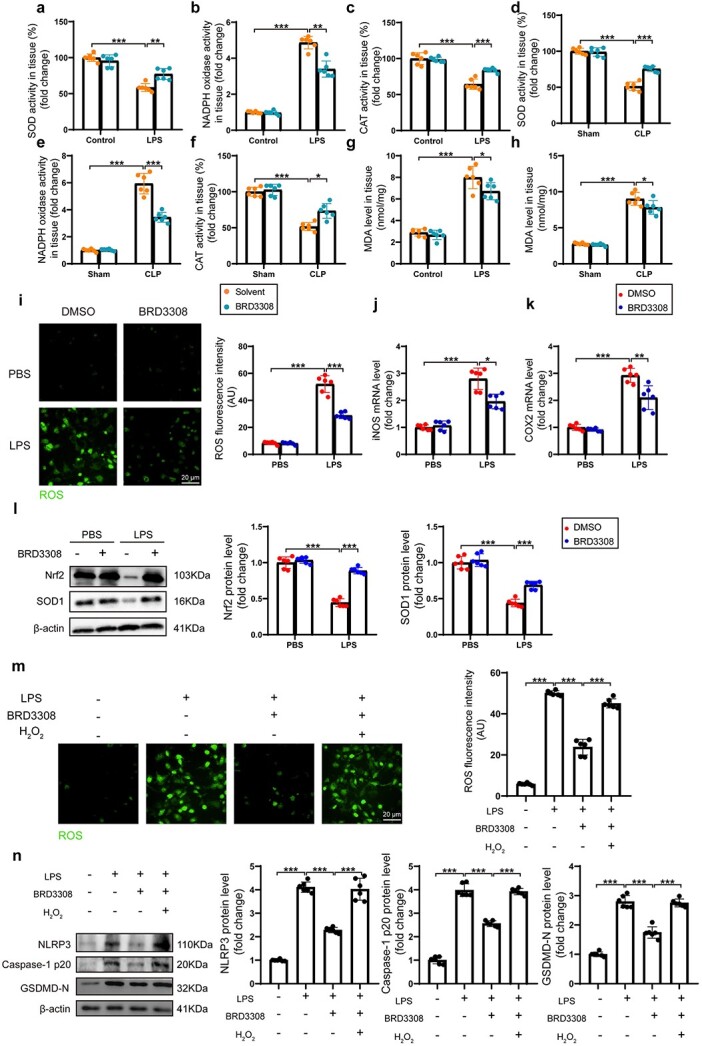
BRD3308 down-regulated macrophage pyroptosis via inhibiting oxidative stress. (**a**–**h**) Assessment of pulmonary tissue oxidative stress levels from mice in the indicated groups was conducted by measuring the activities of SOD, NADPH oxidase (NOX) and CAT, and the level of MDA (n = 6). (**i**) Levels of reactive oxygen species (ROS) in THP-1 cells subjected to different treatments (n = 6, Scale bar: 20 μm). (**j**,**k**) mRNA level of iNOS and COX2 in THP-1 cells was obtained through real-time PCR detection (n = 6). (**l**) Western blot images and data analysis to demonstrate the expression levels of Nrf2 and SOD1 in the THP-1 cells (n = 6). (**m**) Levels of ROS in THP-1 cells from the indicated groups (n = 6, Scale bar: 20 μm). (**n**) Western blot images and data analysis to demonstrate the expression levels of NLRP3, caspase-1 p20 and GSDMD-N in the THP-1 cells (n = 6). The data are presented as mean ± standard deviation (^*^p < 0.05, ^*^^*^p < 0.01, ^*^^*^^*^p < 0.001 compared with indicated group). *iNOS* inducible nitric oxide synthase, *SOD* superoxide dismutase, *NADPH* nicotinamide adenine dinucleotide phosphate, *CAT* catalase, *THP-1* Tamm-Horsfall protein-1,* LPS* lipopolysaccharide, *PBS* Phosphate-Buffered Saline

Sepsis often induces severe oxidative stress, and the accompanying accumulation of ROS is a crucial factor in the increase in NLRP3 expression and activation. To further confirm whether BRD3308 attenuates oxidative stress in sepsis-induced ALI, the activities of oxidative-reduction-related enzymes were assessed in mouse lung tissue. During the onset of sepsis, the enzyme activities of SOD and CAT were significantly decreased, but BRD3308 restored the activity of these enzymes. NADPH oxidase, which exacerbates the disruption of redox balance, exhibited a significant increase under LPS stimulation. However, BRD3308 activity was markedly suppressed when BRD3308 was administered in advance (Figure 4 a-f). Similarly, BRD3308 alleviated the generation of the cellular oxidative injury marker MDA in lung tissue during sepsis occurrence (Figure 4g-h), which demonstrated that BRD3308 exerted an antioxidative effect on sepsis-induced redox imbalance in pulmonary tissue. As expected, the ROS levels were increased in THP-1 cells induced by LPS, and this increase was attenuated by BRD3308 (Figure 4i). The mRNA levels of two pivotal enzymes, iNOS and COX-2, which are known for their positive effects on oxidative stress, were assessed. BRD3308 exerted a negative regulatory influence on the transcription of these enzymes, which suggested that BRD3308 participated in modulating redox stability in LPS-stimulated macrophages (Figure 4j and k). Under the influence of BRD3308, the levels of Nrf2 and SOD1, which are key proteins involved in the antioxidant response, were significantly restored (Figure 4l). Hence, elucidating the interplay between oxidative stress and pyroptosis when BRD3308 is active constitutes an essential approach for in-depth elucidation of the mechanism of action of BRD3308. Combining prior research with the present findings suggested that ROS, which act as damage-associated molecular patterns when present at aberrantly high levels, enhanced NLRP3 inflammasome-mediated pyroptosis in macrophages (Figure S3a, see online supplementary material). Conversely, the mitigation of ROS levels led to a significant reduction in pyroptosis (Figure S3b, see online supplementary material). Following oxidative stress activation induced by H_2_O_2_ in THP-1 cells, the antioxidant capacity of BRD3308 was decreased (Figure 4m). Moreover, the inhibitory effect of BRD3308 on pyroptosis was significantly reversed by H_2_O_2_ (Figure 4n). In summary, the anti-inflammatory function of BRD3308 relies on the clearance of ROS.

### BRD3308 clears ROS through the activation of autophagy in LPS-stimulated macrophages

**Figure 5:**
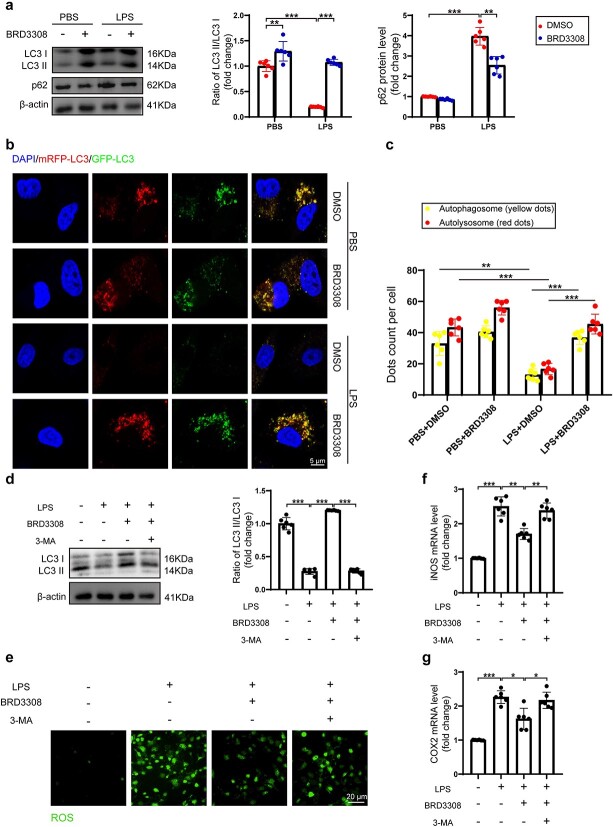
BRD3308 cleared ROS through the activation of autophagy in lipopolysaccharide (LPS)-stimulated macrophages. (**a**) Western blot images and data analysis to demonstrate the expression levels of LC3 I/II and p62 in THP-1 cells (n = 6). (**b**,**c**) Representative fluorescence images and data analysis of THP-1 cells expressing mRFP-GFP-LC3 protein were obtained through confocal microscopy (n = 6, Scale bar: 5 μm). (**d**) Western blot images and data analysis to demonstrate the expression levels of LC3 I/II in the THP-1 cells (n = 6). (**e**) Levels of ROS in THP-1 cells from the indicated groups (n = 6, Scale bar: 20 μm). (**f**,**g**) mRNA levels of iNOS and COX2 in THP-1 cells were obtained through real-time PCR detection (n = 6). The data are presented as mean ± standard deviation (^*^p < 0.05, ^*^^*^p < 0.01, ^*^^*^^*^p < 0.001 compared with indicated group). *ROS* reactive oxygen species, *THP-1* Tamm-Horsfall protein-1, *iNOS* inducible nitric oxide synthase, *PBS* Phosphate-Buffered Saline

Autophagy is one of the crucial pathways for clearing ROS [26]. Thus, the present study investigated whether BRD3308 suppresses ROS levels via modulating the autophagic flux by assessing the protein levels of the LC3 I/II and p62 autophagic markers in THP-1 cells using western blot analysis. BRD3308 effectively restored the LC3 II/I ratio and increased p62 degradation (Figure 5a). After transducing THP-1 cells with a lentivirus carrying mRFP-GFP-LC3, alterations in autophagic flux were observed via confocal microscopy. Both the number of autophagosomes, represented by yellow dots, and the number of autolysosomes, represented by red dots, were significantly increased upon treatment with BRD3308 (Figure 5b and c). Taken together, these results indicated that BRD3308 promoted autophagy in LPS-stimulated THP-1 cells. Next, 3-MA was used to inhibit autophagy, which markedly enhanced the ROS accumulation induced by LPS in THP-1 cells, counteracting the antioxidant capacity of BRD3308 (Figure 5d and e). Additionally, the mRNA levels of *inos* and *Cox2* in THP-1 cells were similarly elevated by 3-MA (Figure 5f and g). Overall, these findings demonstrated that promoting autophagy is a dominant mechanism underlying the antioxidant effect of BRD3308.

### BRD3308 promotes autophagy to inhibit pyroptosis in sepsis-induced ALI

**Figure 6:**
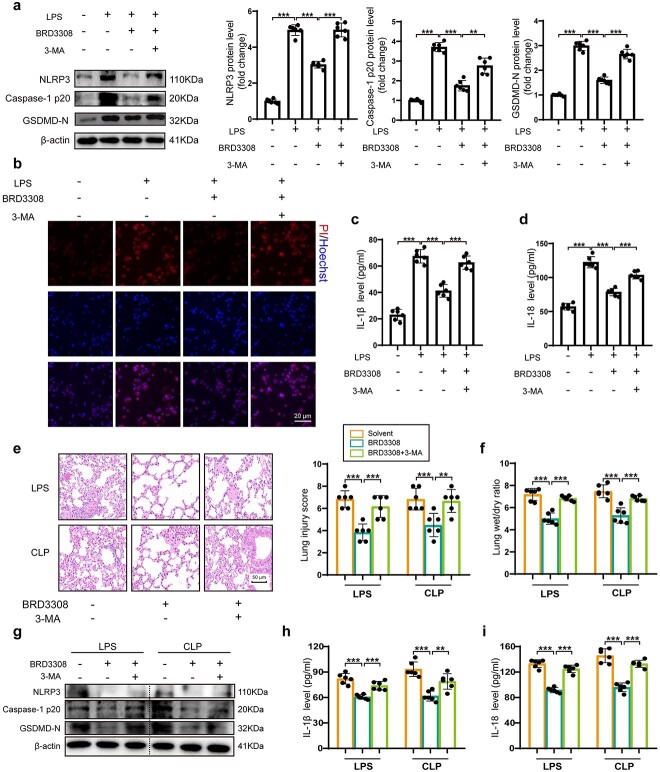
BRD3308 relied on autophagy to inhibit pyroptosis in sepsis-induced acute lung injury (ALI). (**a**) Western blots images and data analysis to demonstrate the expression levels of NLRP3, caspase-1 p20 and GSDMD-N in THP-1 cells (n = 6). (**b**) Representative images of PI & Hoechst staining in THP-1 cells from the indicated groups (n = 3, Scale bar: 20 μm). (**c**,**d**) Activity levels of interleukin (IL)-1β and IL-18 in cell supernatant were determined by ELISA (n = 6). (**e**) Hematoxylin and eosin (H&E) staining images and injury scores of lung tissue sections from mice in the indicated groups (n = 6, Scale bar: 50 μm). (**f**) Lung wet-to-dry ratio of mice from the indicated groups (n = 6). (**g**) Western blots images and data analysis to demonstrate the expression levels of NLRP3, Caspase-1 p20 and GSDMD-N in treated mouse alveolar macrophages (n = 6). (**h**,**i**) Activity levels of IL-1β and IL-18 in lung tissue were determined by ELISA (n = 6). The data are presented as mean ± standard deviation (^*^^*^p < 0.01, ^*^^*^^*^p < 0.001 compared with indicated group). *CLP *cecal ligation and puncture, *ELISA* enzyme-linked immunosorbent assay, *IL* interleukin, *NLRP3* Nod-like receptor protein 3, *GSDMD* gasdermin-D, *THP-1* Tamm-Horsfall protein-1

To further validate the protective effect exerted by BRD3308 in positively modulating macrophage autophagy in response to LPS stimulation, THP-1 cells were cotreated with BRD3308 and the autophagy inhibitor 3-MA. In THP-1 cells cotreated with BRD3308 and 3-MA after LPS stimulation, the protein levels of NLRP3, caspase-1 p20 and GSDMD-N were significantly higher than those in cells treated with BRD3308 alone (Figure 6a). PI and Hoechst staining demonstrated that the protective effect of BRD3308 on macrophage membrane integrity was compromised by 3-MA treatment (Figure 6b). After autophagy inhibition, the activation of IL-18 and IL-1β was also significantly elevated (Figure 6c and d). These results suggested that the antipyroptotic effect of BRD3308 on LPS-challenged THP-1 cells is compromised by autophagy inhibition. To verify these results *in vivo*, septic mice were administered intraperitoneal injections of 3-MA to inhibit autophagy. The pathological findings indicated that mice in the BRD3308 + 3-MA group exhibited more severe lung tissue damage, and no improvement in pulmonary edema was observed (Figure 6e and f). The protein levels of pyroptosis markers in mouse alveolar macrophages and the levels of secreted IL-18 and IL-1β in lung tissues were assessed (Figure 6g–i). These findings further supported the conclusion that BRD3308 regulates pyroptosis via autophagic activation.

### BRD3308 exerts a protective effect by selectively inhibiting the enzymatic activity of HDAC3

**Figure 7:**
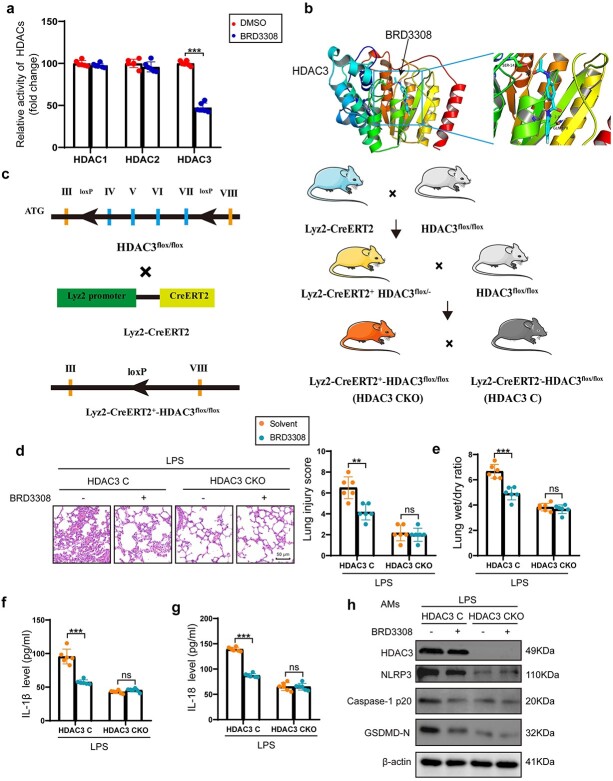
BRD3308 exerted a protective effect by selectively inhibiting the enzymatic activity of histone deacetylase 3 (HDAC3). (**a**) HDACs enzyme activity in THP-1 cells treated with BRD3308 was measured by fluorescence assay kits (n = 6). (**b**) Molecular docking showed binding domain of BRD3308 and human HDAC3. Predicted binding sites: Ser-142, Gln-178. (**c**) Schematic representation of the generation of HDAC3 conditional knockout (CKO) mice in myeloid cells. (**d**) Hematoxylin and eosin (H&E) staining images and injury scores of lung tissue sections corresponding to mice in the intraperitoneally injected lipopolysaccharide (LPS) modeling groups with or without BRD3308 treatment (n = 6, Scale bar: 50 μm). (**e**) Lung wet-to-dry ratio was obtained after weighing the wet and dry weights of the lung tissues from indicated groups of mice (n = 6). (**f**,**g**) Activity levels of IL-1β and IL-18 in lung tissue were determined by ELISA (n = 6). (**h**) Western blot images and data analysis to demonstrate the expression levels of HDAC3, NLRP3, Caspase-1 p20 and GSDMD-N in treated mouse alveolar macrophages (n = 6). The data are presented as mean ± standard deviation (^*^^*^p < 0.01, ^*^^*^^*^p < 0.001 compared with indicated group, ns, no significance). *ELISA* enzyme-linked immunosorbent assay, *IL* interleukin, *NLRP3* Nod-like receptor protein 3, *GSDMD* gasdermin-D, *THP-1* Tamm-Horsfall protein-1, *HDAC* histone deacetylase

After confirming the protective effect of BRD3308 against sepsis-induced ALI, the key target of BRD3308 was further investigated. Previous studies have reported that BRD3308 is a specific inhibitor of HDACs, and its inhibitory effects on HDAC1, HDAC2 and HDAC3 vary at different concentrations. In the present study, the enzymatic activity of class I HDACs was examined after treatment with the desired concentration of BRD3308 in the culture medium of THP-1 cells. BRD3308 inhibited the enzymatic activity of HDAC3 in THP-1 cells (Figure 7a). Molecular docking analysis predicted that BRD3308 may bind and exert its function at two sites, namely, S142 and G178, within HDAC3 (Figure 7b). Because these two sites are not located within the reported catalytic domain of HDAC3, BRD3308 may influence the activity of HDAC3 by altering its protein conformation. Mice with specific knockout of *Hdac3* in macrophages were generated and utilized to elucidate the role of BRD3308 (Figure 7c). *Hdac3* floxed mice and *Hdac3* conditional knockout (*Hdac3* cKO) mice were separately administered intraperitoneal injections of BRD3308 for seven consecutive days, followed by LPS-induced modeling. Pathology revealed that BRD3308 had a pronounced protective effect on lung tissue damage in control mice (Figure 7d). However, for mice lacking HDAC3, the administration of BRD3308 did not yield additional improvements in lung tissue (Figure 7d). The extent of pulmonary tissue edema in the mice showed a similar pattern (Figure 7e). Macrophage pyroptosis was assessed (Figure 7f–h), which indicated that BRD3308 did not further inhibit pyroptosis in *Hdac3* cKO mice. Therefore, the *in vivo* and *in vitro* experimental results suggested that BRD3308 primarily improves ALI by inhibiting the enzymatic activity of HDAC3 in macrophages.

### BRD3308 upregulates ATG5 expression by promoting H3K27 acetylation

**Figure 8:**
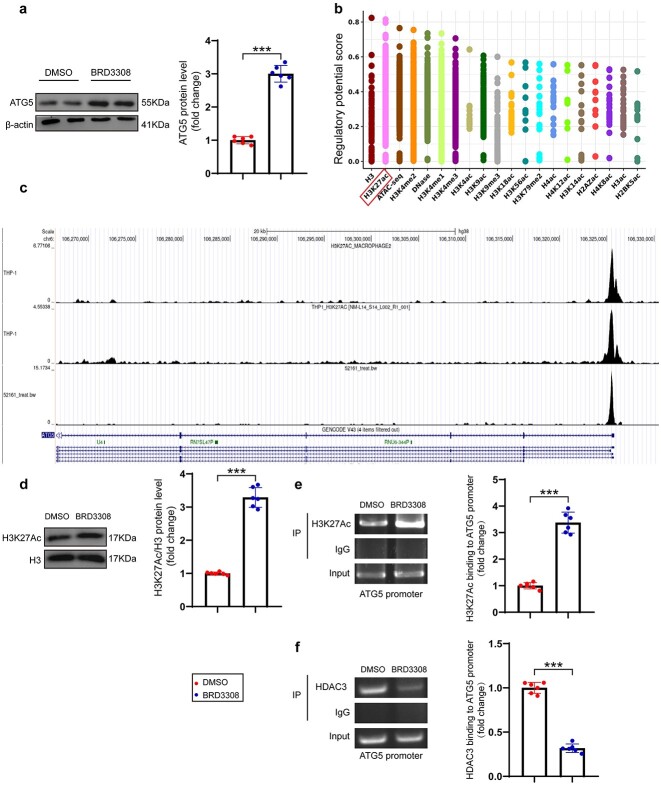
BRD3308 upregulated autophagy-related gene 5 (ATG5) expression via promoting H3K27 acetylation. (**a**) Western blot images and data analysis to demonstrate the expression level of ATG5 in THP-1 cells (n = 6). (**b**) Scoring of the analysis results for different histone modification sites binding to the ATG5 promoter to regulate ATG5 expression. (**c**) Analysis of the binding sites of H3K27Ac to the ATG5 gene in THP-1 cells based on three chromatin immunoprecipitation (ChIP)-seq datasets from the UCSC genome browser. (**d**) Western blot images and data analysis to demonstrate the impact of BRD3308 on the acetylation levels of H3K27 in THP-1 cells (n = 6). (**e**,**f**) Changes in the binding levels of HDAC3 or H3K27Ac with the ATG5 promoter after BRD3308 intervention were analyzed through ChIP experiments (n = 6). The data are presented as mean ± standard deviation (^*^^*^^*^p < 0.001 compared with indicated group). *DMSO* dimethyl sulfoxide, *HDAC3* Histone deacetylase 3, *THP-1* Tamm-Horsfall protein-1

Histone acetylation regulates the expression of the majority of genes, and HDAC3 is a key enzyme in histone deacetylation. The mechanism of action of BRD3308 was explored by focusing on the intrinsic function of HDAC3. First, the protein level of the ATG5 key autophagy regulator was assessed after treatment with BRD3308 (Figure 8a), which demonstrated that BRD3308 induced an increase in the expression of ATG5. Subsequently, Cistrome DB (http://cistrome.org/db/#/) was utilized to predict histone sites that may bind to the promoter of *Atg5* and potentially regulate its expression. Acetylation at H3K27 had the highest probability of regulating the *Atg5* promoter (Figure 8b). Furthermore, datasets for THP-1 cells or macrophages were utilized to predict potential binding sites of H3K27 to the *Atg5* promoter. Figure 8c displays highly similar H3K27ac binding sites to the *Atg5* promoter across different datasets, providing further confirmation of the predicted outcome. Western blot analysis demonstrated that BRD3308 altered the acetylation levels of H3K27 in THP-1 cells (Figure 8d). Chromatin immunoprecipitation was used to separately assess the binding capacities of HDAC3, H3K27ac and the *Atg5* promoter. BRD3308 intervention enhanced the binding of H3K27ac to the *Atg5* promoter, but it inhibited the binding of HDAC3 to the *Atg5* promoter (Figure 8e and f). Previous studies have shown that histone acetylation promotes the expression of corresponding genes at specific loci by loosening chromatin structure. Therefore, the present findings suggest that BRD3308 promotes H3K27 acetylation in macrophages by inhibiting the activity of HDAC3, thereby facilitating the expression of ATG5.

### ATG5 is the crucial target of BRD3308 in inhibiting macrophage pyroptosis

**Figure 9:**
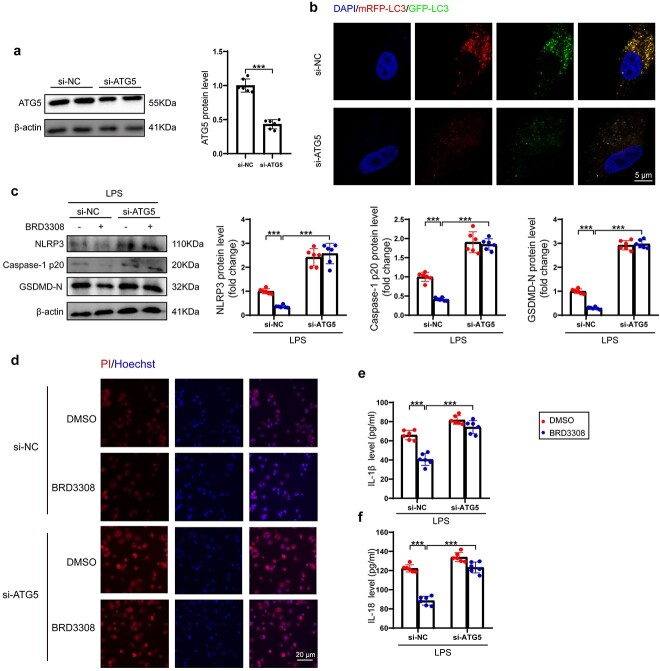
Autophagy-related gene 5 (ATG5) was the crucial target of BRD3308 in inhibiting macrophage pyroptosis. (**a**) Western blot images and data analysis to demonstrate the expression level of ATG5 in THP-1 cells (n = 6). (**b**) Representative fluorescence images of THP-1 cells expressing mRFP-GFP-LC3 protein were obtained through confocal microscopy (n = 3, Scale bar: 5 μm). (**c**) Western blot images and data analysis to demonstrate the expression levels of NLRP3, caspase-1 p20 and GSDMD-N in THP-1 cells (n = 6). (**d**) Representative images of PI & Hoechst staining in THP-1 cells from the indicated groups (n = 3, Scale bar: 20 μm). (**e**,**f**) Activity levels of IL-1β and IL-18 in cell supernatant were determined by ELISA (n = 6). The data are presented as mean ± standard deviation (^*^^*^^*^p < 0.001 compared with indicated group). *ELISA* enzyme-linked immunosorbent assay,* IL* interleukin, *NLRP3* Nod-like receptor protein 3, *GSDMD* gasdermin-D, *THP-1* Tamm-Horsfall protein-1, *LPS* lipopolysaccharide

After confirming that ATG5 was upregulated by BRD3308, the present study investigated whether ATG5 has sufficient antipyroptotic effects on macrophages. In THP-1 cells transfected with si-ATG5, the protein levels of ATG5 were significantly reduced and autophagic flux was markedly inhibited (Figure 9a and b). In THP-1 cells treated with either DMSO or BRD3308 and stimulated with LPS, ATG5 deficiency significantly increased the protein levels of NLRP3, caspase-1 p20 and GSDMD-N (Figure 9c). This effect significantly counteracted the inhibitory effect of BRD3308 on the protein levels of these three factors. Similarly, knockdown of ATG5 significantly increased the extent of cell membrane damage, even after prior intervention with BRD3308 (Figure 9d). Furthermore, the levels of secreted IL-1β and IL-18 in the cell supernatant increased with reduced expression of ATG5, demonstrating a substantial attenuation of the pharmacological effect of BRD3308 (Figure 9e and f). In conclusion, these results indicate that the upregulation of Atg5 expression is a crucial factor in the protective effect of BRD3308 on sepsis-induced ALI.

## Discussion

Sepsis is associated with high morbidity and mortality worldwide. Intractable hypoxemia due to sepsis-induced ALI is one of the key causes of the high lethality of sepsis. Our previous research has confirmed that macrophage pyroptosis disrupts immune microenvironment stability in sepsis-induced ALI, leading to an inflammatory cytokine storm and further damage to the alveolar structure [18]. In contrast, inhibiting pyroptosis results in improvements in both inflammation and tissue injury in sepsis-induced ALI [27]. Despite the increasing number of studies dedicated to exploring the upstream mechanisms of macrophage pyroptosis and targeted therapeutic strategies in ALI, there are still significant gaps of knowledge. Therefore, the present study focused on the regulatory targets of pyroptosis in macrophages to validate the protective effect of the BRD3308 class I HDAC inhibitor on ALI, as well as its inhibitory effect on pyroptosis. After fully elucidating the protective role of BRD3308, the present study further demonstrated its inhibitory effect on the enzymatic activity of HDAC3. Moreover, BRD3308 maintained redox stability under LPS stimulation by upregulating the acetylation of H3K27 and enhancing autophagic flux, ultimately reducing NLRP3-mediated macrophage pyroptosis. From the perspective of histone acetylation regulation, the present study systematically verified the ameliorative effect of BRD3308 on sepsis-induced ALI and its potential mechanism, providing a theoretical basis for clinically protecting pulmonary function in septic patients.

The expression of histones in eukaryotic cells is relatively stable and less susceptible to external influences. However, alterations in histone modification levels can have a significant impact on most cellular life processes. Many studies have indicated that histone modifications in different cell types play varying roles in the development and progression of lung injury. H3K4 trimethylation (H3K4me3) exerts a protective effect by upregulating the expression of keratinocyte growth factor-2, thereby improving endothelial barrier disruption caused by lung injury [28]. In macrophages, however, trimethylation at H3K36 (H3K36me3) alleviates ALI by promoting the expression of SET domain-containing 2, which in turn inhibits glycolysis [29]. Our previous study has demonstrated that acetylation of H3K9 in macrophages mitigates inflammation and damage during ALI by inhibiting the cGAS-STING pathway [18]. Histone acetylation, a pivotal epigenetic modification, is dynamically regulated through the antagonistic actions of HATs and HDACs. HATs catalyze the transfer of acetyl groups from acetyl-CoA to lysine residues on histones, thereby promoting the relaxation of chromatin structure and enhancing the transcriptional activation of associated genes [30]. Conversely, HDACs work in opposition to this process by removing acetyl groups from histones, leading to a reduction in the expression level of the corresponding genes [31]. HDAC3, an HDAC capable of shuttling between the nucleus and cytoplasm, not only regulates histone deacetylation but also governs the deacetylation of nonhistone proteins within the cytoplasm [32]. The stable and extensive protein-binding capacity of HDAC3 ensures that it plays a crucial regulatory role in the growth, development and even injury of various vital organs. Yao *et al*. reported that HDAC3 orchestrates the PPAR-γ signaling cascade by augmenting Pparg expression, consequently fine-tuning the growth and development of alveolar macrophages [33]. Additionally, HDAC3 plays an epigenetic modulatory role in regulating the miR-17-92/TGF-β pathway, thereby impacting the early formation of pulmonary alveoli [34]. The functions of HDAC3 in pulmonary diseases are gradually being elucidated. Under hypoxic conditions, HDAC3 stabilizes HIF-1α through the AKT pathway, promoting hypoxia-induced alveolar epithelial–mesenchymal transition and enhancing fibroblast migration and invasion [35]. Additionally, numerous studies have confirmed that *Hdac3* is a risk gene exacerbating pulmonary fibrosis [36]. Our group has investigated the role and related mechanisms of HDAC3 in ALI. In ALI, HDAC3 in type II alveolar epithelial cells disrupts mitochondrial function by upregulating ROCK1 expression through the deacetylation of FOXO1 [17]. In macrophages, HDAC3 induces histone deacetylation to promote pyroptosis, contributing to the development of ALI [18]. Based on these findings, future studies will focus on identifying small molecules capable of selectively inhibiting HDAC3 enzymatic activity, with the aim of providing potential clinical applications.

To date, various HDAC inhibitors have been employed in clinical settings and have demonstrated therapeutic efficacy [37]. The nonselective inhibitor of class I and class II HDACs, vorinostat, is the first HDAC inhibitor approved for the treatment of cancer [38]. When used as a monotherapy, vorinostat has demonstrated favorable therapeutic effects in various solid and hematologic malignancies, including head and neck cancer, diffuse large B-cell lymphoma, glioblastoma multiforme, Hodgkin’s lymphoma and even myelodysplastic syndrome [38]. Moreover, the use of vorinostat in combination with chemotherapeutic agents, such as cisplatin, results in significant synergistic activity [39]. Other inhibitors, such as panobinostat, tucidinostat and apalutamide, have garnered widespread recognition in the clinical or preclinical stages for treating various cancers and immune-system disorders [40–42]. In recent years, an increasing body of research has indicated that HDAC inhibitors, such as tubastatin and vorinostat, play a significant role in ameliorating pulmonary fibrosis by correcting aberrant acetylation [43,44]. Another nonselective inhibitor, butyrate, has been reported to alleviate sepsis-induced ALI by inhibiting p65 activation and, to some extent, mitigating the level of inflammation [45]. However, for sepsis-induced ALI, there is still a lack of highly selective and targeted HDAC inhibitors, representing an opportunity for further research. In the present study, the preventive and therapeutic effects of a relatively novel class I HDAC inhibitor, BRD3308, on sepsis-induced ALI were validated. Together, these findings suggest that although BRD3308 inhibits both HDAC1 and HDAC2 to a certain extent, it demonstrates strong selectivity toward HDAC3 at lower concentrations, significantly suppressing HDAC3 enzymatic activity. In septic mice lacking HDAC3, BRD3308 does not exhibit additional protective effects, further confirming its pharmacological dependency on HDAC3. Additionally, the predicted binding sites of BRD3308 with HDAC3 are S142 and G178, which are not located within the active site of HDAC3. Thus, these findings suggest that BRD3308 affects the enzymatic activity of HDAC3 by altering its protein conformation through binding interactions.

LPS, a potent inflammatory stimulus, exacerbates the synthesis of proinflammatory mediators and ROS upon recognition. The mechanism by which LPS exacerbates oxidative stress is relatively intricate. LPS not only activates ROS-generating enzymes, such as NADPH oxidase, and inhibits antioxidant defense mechanisms but also disrupts the electron transport chain within mitochondria to augment ROS production [46,47]. An increasing body of research emphasizes the pivotal role of oxidative stress in promoting the onset of inflammation in sepsis-induced acute organ injury. Autophagy, a highly conserved biological process, responds to damage signals by forming autophagosomes to encapsulate and clear aberrant or damaged organelles and proteins [48]. This represents one of the most crucial pathways for maintaining cellular redox homeostasis. Research has indicated that autophagy inhibits the synthesis and accumulation of ROS by modulating redox-related signaling pathways [49]. Moreover, autophagy identifies and removes oxidatively damaged organelles, such as mitochondria, to reduce the amount of ROS produced [50]. Based on this theoretical foundation, BRD3308 amplifies the autophagic flux during sepsis induction to effectively clear intracellular ROS, thereby inhibiting macrophage pyroptosis.

ATG5 is a crucial protein in the autophagy process that plays a significant role in the formation of autophagosomes [51]. ATG5 initially associates with ATG12 to form the ATG5–ATG12 complex, a pivotal step in the autophagic process. Subsequently, the ATG5–ATG12 complex binds with another protein, ATG16L, forming the ATG5–ATG12–ATG16L complex. This complex aids in membrane expansion and closure, ultimately culminating in complete autophagosome formation [52]. The protective role of ATG5-mediated autophagy has been confirmed in various organ injuries. In acute kidney injury, ATG5-dependent autophagy alleviates renal damage by inhibiting the activation of p65 [53]. Similarly, in liver injury, ATG5 protects against damage by suppressing the proinflammatory polarization of Kupffer cells [54]. Given the dual-edged nature of autophagy, excessive autophagy mediated by ATG5 has also been demonstrated to exacerbate brain injury by upregulating ferroptosis [55]. In the present study, BRD3308 counteracted the early suppression of autophagy induced by LPS. Therefore, these findings suggested that BRD3308 may upregulate a key gene during the autophagosome formation stage. Through prediction and experimental validation, the present study confirmed that BRD3308 promoted the expression of ATG5. Considering the fundamental function of HDAC3, the histone site corresponding to the ATG5 promoter was predicted, and its modification level was assessed. BRD3308 effectively inhibited the activity of HDAC3, leading to an increase in H3K27ac levels and subsequently promoting the transcriptional activation of ATG5.

The present study had several limitations. The study focused primarily on the regulation of histone acetylation in macrophages by BRD3308, with HDAC3 as the main target. Although the important role of HDAC3 was elucidated in this process through *in vitro* and *in vivo* experiments and the interaction was successfully predicted, the precise binding sites of BRD3308 with HDAC3 still require further experimental validation. Moreover, extensive research has established that the regulatory effects of HDAC3 extend beyond histone deacetylation, as it also influences the acetylation, lactylation and succinylation of nonhistone proteins, among various modifications [17,56,57]. Therefore, exploring the broader effects of BRD3308 through modification omics (modomics) will provide a theoretical basis for its accelerated clinical application.

## Conclusions

In conclusion, the present study elucidated the significant protective role of BRD3308 in sepsis-induced ALI. Specifically, BRD3308 inhibited the enzymatic activity of HDAC3 in macrophages, leading to the upregulation of acetylation at the H3K27 histone site and promoting transcriptional activation of the corresponding promoter. Subsequently, prediction and experimental validation indicated that ATG5 was significantly elevated during this process, facilitating autophagy and suppressing the accumulation of ROS to maintain redox homeostasis, ultimately downregulating macrophage pyroptosis. Thus, the present study not only highlights the promising therapeutic potential of BRD3308 in sepsis-induced ALI but also emphasizes the regulatory role of HDAC3 in its pathogenesis. The present findings provide a fresh perspective and a solid theoretical foundation for the clinical prevention of sepsis-induced ALI.

## Supplementary Material

Supplement_Figures_tkae033

## Data Availability

All data that support the findings in this study are available from the corresponding author upon reasonable request.

## Abbreviations

ALI: Acute lung injury; ATG5: Autophagy-related gene 5; CLP: Cecal ligation and puncture; GSDMD: Gasdermin-D; HATs: Histone acetyltransferases; HDACis: HDAC inhibitors; HDACs: Histone deacetylases; IL-1β: Interleukin-1β; LPS: Lipopolysaccharide; NLRP3: Nod-like receptor protein 3; ROS: Reactive oxygen species, 3­MA: 3-Methyladenine, MPO: myeloperoxidase, SOD: superoxide dismutase, CAT: catalase, MDA: malondialdehyde, PI: propidium iodide, FITC: fluorescein isothiocyanate ,DAPI: 4',6-diamidino-2-phenylindole, PBS: Phosphate-Buffered Saline, DMEM: Dulbecco's Modified Eagle Medium, FBS: fetal bovine serum, PMA: Phorbol 12-myristate 13-acetate, CCK­8: Cell Counting Kit-8, EDTA: ethylenediaminetetraacetic acid, BCA: bicinchoninic acid, PVDF: polyvinylidene fluoride, ELISA: enzyme-linked immunosorbent assay, iNOS: inducible nitric oxide synthase, COX­2: cyclooxygenase-2, PPAR­γ : peroxisome proliferator-activated receptors γ, Pparg: proliferator-activated receptor gamma, AKT: protein kinase B, FOXO1: forkhead box O1, HIF­1α: hypoxia-inducible factor-1α, MCP­1: monocyte chemoattractant protein-1, TNF­α: tumor necrosis factor­α, DMSO: dimethyl sulfoxide, DCFH­DA: 2’,7’-dichlorodihydrofluorescein diacetate.
