# Supplementary material for: BRD3308 suppresses macrophage oxidative stress and pyroptosis via upregulating acetylation of H3K27 in sepsis-induced acute lung injury
Source: Burns Trauma. 2024 Sep 2;12:tkae033. doi: 10.1093/burnst/tkae033 (PMC11367671; doi:10.1093/burnst/tkae033)
Supplement: Supplement_Figures_tkae033 [file supplement_figures_tkae033.docx]

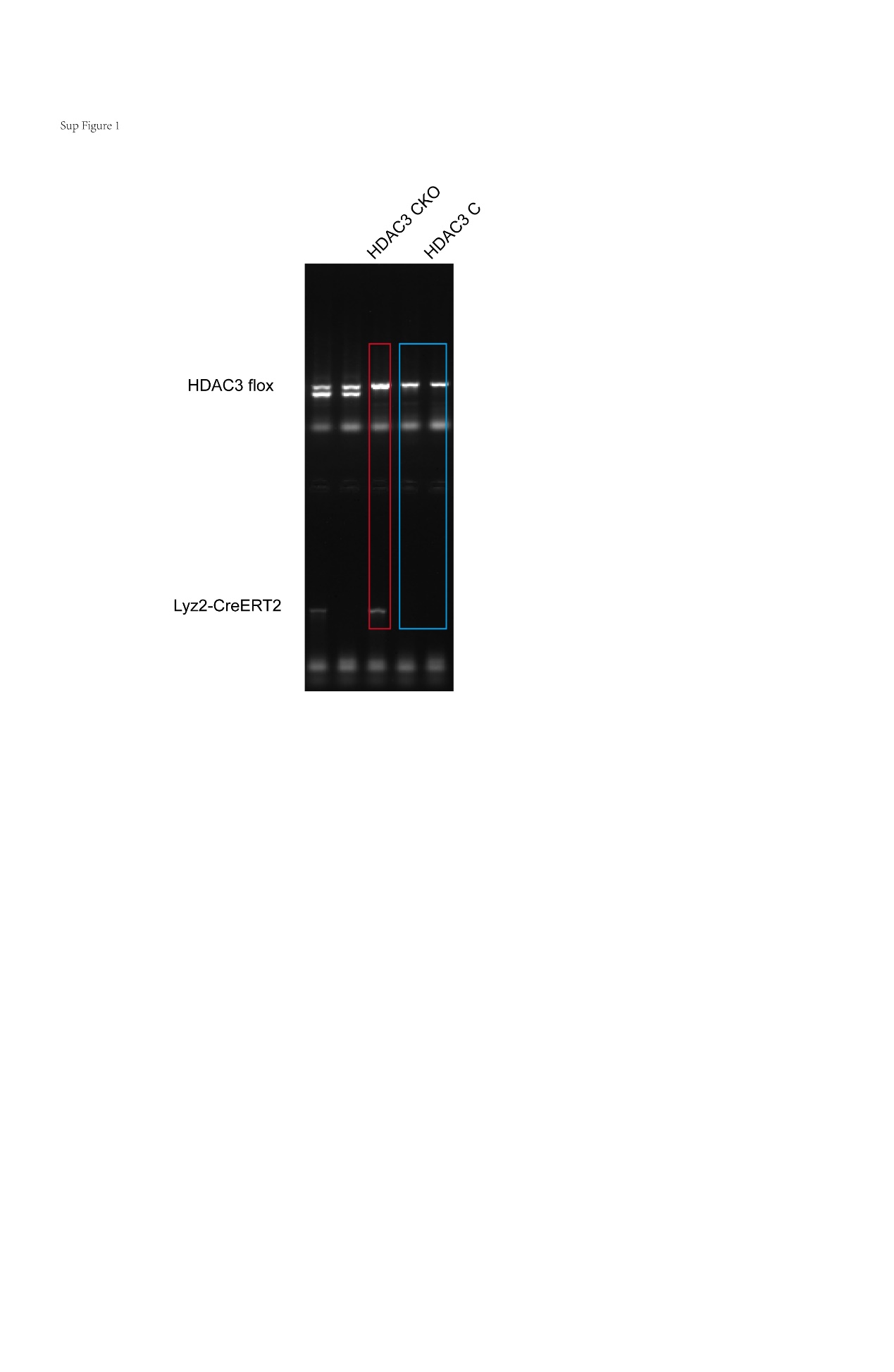


**Figure S1.** The agarose gel electrophoresis showing the genotype identification results of HDAC3 CKO and HDAC3 C mice


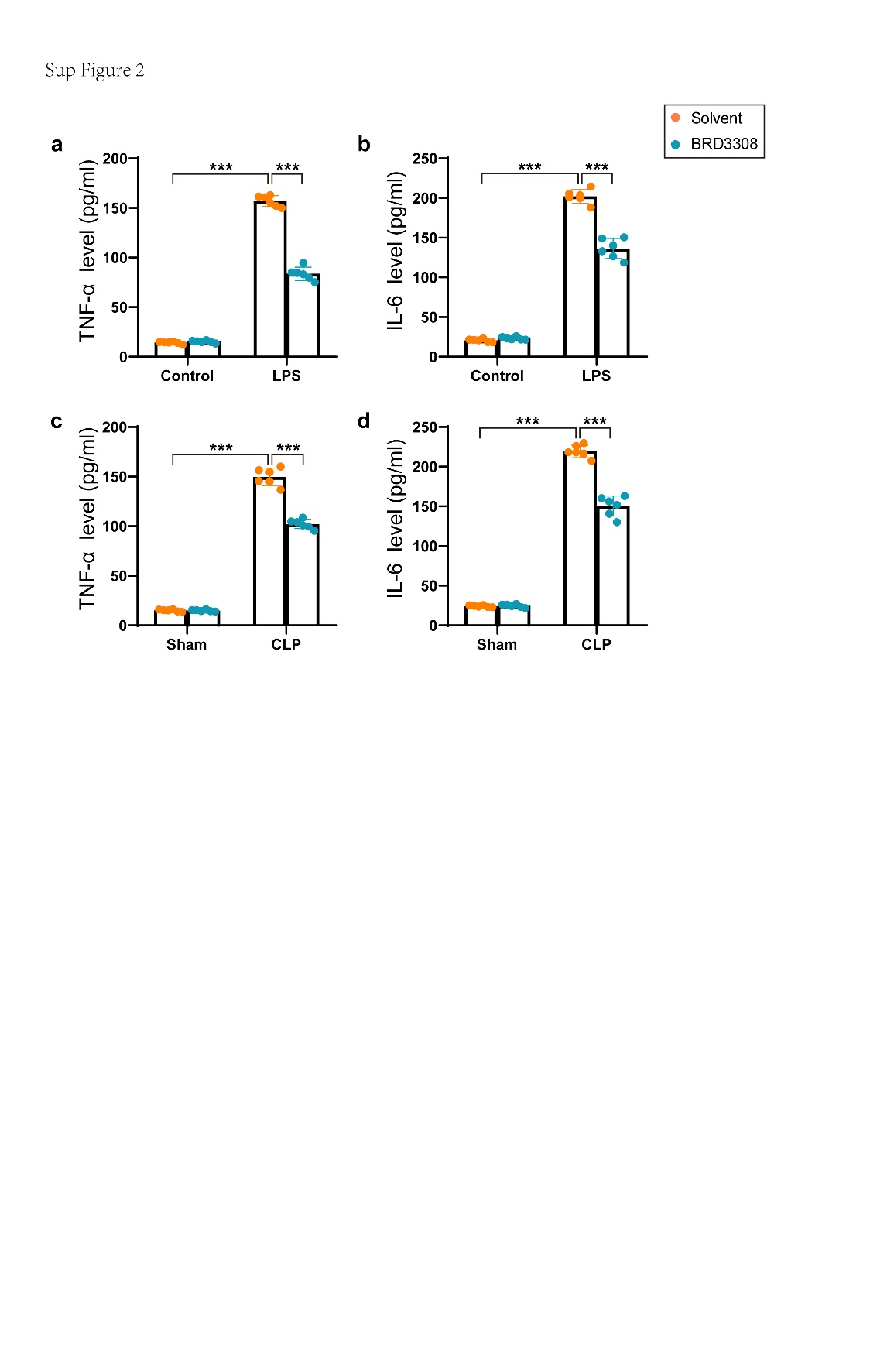


**Figure S2. BRD3308 significantly suppressed the release levels of** **TNF-α and IL-6 in the pulmonary tissue of septic mice. (a-d)** The activity levels of TNF-α and IL-6 in lung tissue were determined by ELISA (n=6). The data was presented as mean ± standard deviation (*** *p* <0.001 compared with indicated group, ns, no significance). *TNF-α* tumor necrosis factor, *ELISA* enzyme-linked immunosorbent assay, *IL* interleukin


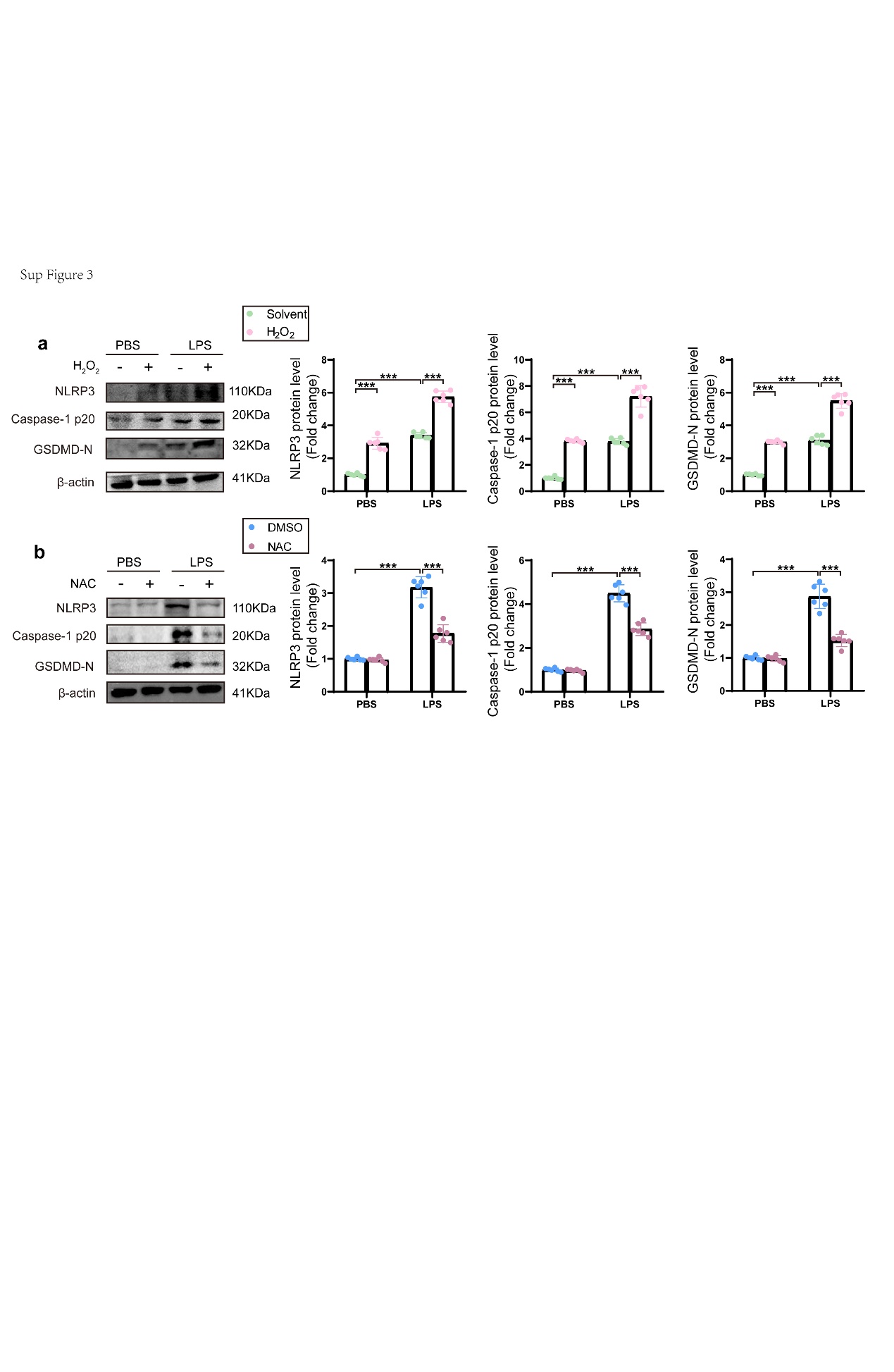


**Figure S3. ROS was a key factor mediating pyroptosis in macrophages(a-b)** Western blots images and data analysis to demonstrate the expression levels of NLRP3, Caspase-1 p20, and GSDMD-N in the THP-1 cells(n=6). The data was presented as mean ± standard deviation. (*** *p* <0.001 compared with indicated group, ns, no significance). *ROS* reactive oxygen species, *NLRP3* Nod-like receptor protein 3, *GSDMD* gasdermin-D
